# Low-Temperature Direct Growth of Nanocrystalline Multilayer Graphene on Silver with Long-Term Surface Passivation

**DOI:** 10.1021/acsami.2c21809

**Published:** 2023-02-08

**Authors:** Chen-Hsuan Lu, Kuang-Ming Shang, Shi-Ri Lee, Chyi-Ming Leu, Yu-Chong Tai, Nai-Chang Yeh

**Affiliations:** † Department of Applied Physics and Materials Science, California Institute of Technology, Pasadena, California 91125, United States; ‡ Department of Medical Engineering, California Institute of Technology, Pasadena, California 91125, United States; § Department of Electron Microscopy Development and Application, Division of Platform Technology for Advanced Materials, Material and Chemical Research Laboratories, Industrial Technology Research Institute, Hsinchu 31057, Taiwan; ∥ Material and Chemical Research Laboratories, Industrial Technology Research Institute, Hsinchu 31057, Taiwan; ⊥ Department of Electrical Engineering, California Institute of Technology, Pasadena, California 91125, United States; # Department of Physics, California Institute of Technology, Pasadena, California 91125, United States; ¶ Department of Physics, National Taiwan Normal University, Taipei City 106, Taiwan

**Keywords:** PECVD, graphene, low-temperature, silver, passivation

## Abstract

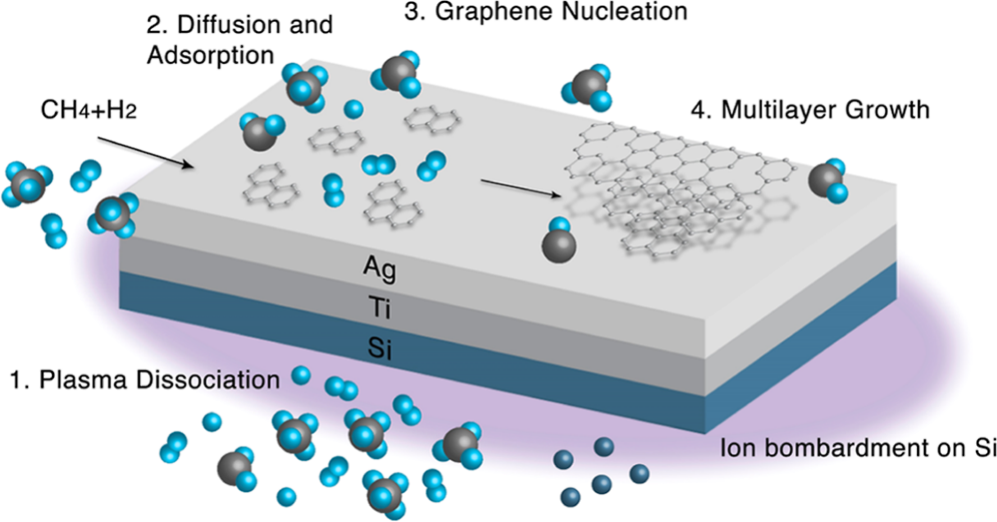

A wide variety of transition metals, including copper and gold, have been successfully used as substrates for graphene growth. On the other hand, it has been challenging to grow graphene on silver, so realistic applications by combining graphene and silver for improved electrode stability and enhanced surface plasmon resonance in organic light-emitting diodes and biosensing have not been realized to date. Here, we demonstrate the surface passivation of silver through the single-step rapid growth of nanocrystalline multilayer graphene on silver via low-temperature plasma-enhanced chemical vapor deposition (PECVD). The effect of the growth time on the graphene quality and the underlying silver characteristics is investigated by Raman spectroscopy, X-ray diffraction, atomic force microscopy, X-ray photoelectron spectroscopy (XPS), and cross-sectional annular dark-field scanning transmission electron microscopy (ADF-STEM). These results reveal nanocrystalline graphene structures with turbostratic layer stacking. Based on the XPS and ADF-STEM results, a PECVD growth mechanism of graphene on silver is proposed. The multilayer graphene also provides excellent long-term protection of the underlying silver surface from oxidation after 5 months of air exposure. This development thus paves the way toward realizing technological applications based on graphene-protected silver surfaces and electrodes as well as hybrid graphene-silver plasmonics.

## 1 Introduction

The unique electronic and optical properties of graphene have stimulated an extensive range of scientific research and technological applications. Additionally, multilayer graphene may be regarded as an alternative to graphite in many graphite-related applications with the benefits of much better scalability in the lateral dimensions^1^ and the much smaller thicknesses. For instance, it has been challenging to produce highly oriented pyrolytic graphite to an areal dimension larger than ∼ (0.1 × 0.1) m^2^. In the case of graphene growth, chemical vapor deposition (CVD) is a common scalable approach to achieve large-scale high-quality graphene synthesis. Common transition-metal substrates such as Ni^2^ and Cu^3^ are used along with high processing temperatures to pyrolyze hydrocarbon molecules to activate CVD graphene growth.^4^ Other transition-metal substrates such as Ag,^5^ Au,^6,7^ Pt,^8^ Ru,^9^ and Co^10^ have been reported for CVD graphene synthesis.

One of the drawbacks of CVD graphene growth is that it is a high-temperature process, typically close to the melting points of the metal foils. Under such conditions, aged quartz furnaces and evaporated metal could lead to contamination.^11^ In addition, substrates involving temperature-sensitive materials such as polymers would be damaged under high-temperature processes, preventing their applications for flexible materials.^12^ Moreover, the current industrial trend of net zero carbon emission by 2050 makes the high-temperature process unfavorable due to high energy consumption. Research progress has been made to reduce the growth temperature for CVD graphene. For instance, a growth temperature of 100 °C has been reported through the use of a benzene precursor.^13^ Moreover, near-room-temperature growth has been demonstrated by utilizing liquid metal nucleation.^14^ Despite the fact that these novel approaches have greatly reduced the growth temperature, they require sophisticated processing steps that are incompatible with industrial processes and large-area production.^13−15^

On the other hand, a scalable and industrially compatible process for low-temperature graphene growth is the plasma-enhanced CVD (PECVD) method. The key factor that allows PECVD to reduce growth temperature lies in the plasma, which contains reactive species to promote growth. Recently, Kim et al.^16^ have further lowered the required temperatures for PECVD growth of graphene by forced convection to increase the reaction probability of excited species or radicals on the substrate surface before their recombination. To date, the reported growth temperatures for various PECVD graphene synthesis methods are found to range from 160 to 700 °C on various substrates.^12,17−22^

Silver is commonly used as the electrode for organic light-emitting diodes and in biosensing due to its strong surface plasmon resonance (SPR). However, silver is prone to oxidation, which would degrade the device performance.^23^ This problem may be mitigated by the combination of graphene with silver, which has been reported to increase the stability of the silver nanowire electrodes^24^ and theoretically predicted to enhance the SPR sensitivity while preventing oxidation.^23^ On the other hand, the inert nature of silver has made it difficult to be used as a substrate for graphene growth with the standard CVD techniques. Among the limited reports of graphene growth on silver, one approach involves evaporating atomic carbon onto the surface of a single-crystalline Ag(111) substrate at elevated temperatures under ultrahigh vacuum conditions for in situ scanning tunneling microscopy studies.^25^ The other approach utilizes a high-temperature, atmospheric-pressure CVD process with solid camphor as the carbon precursor and silver foil as the substrate in a gas mixture of Ar and H_2_.^5^ Despite these progresses, direct graphene growth on silver at low temperatures remains a challenging task.^26^

In this work, we show the viability of direct growth of nanocrystalline multilayer graphene on a silver thin film via low-temperature PECVD, where the growth configuration involves flipping the substrate downward so that the silver thin film faces away from the direct plasma. The successful growth of nanocrystalline multilayer graphene is confirmed by Raman spectroscopy. X-ray diffraction (XRD) studies of the silver thin films after the PECVD process further reveal the improvement of silver crystallinity. Studies by transmission electron microscopy (TEM) reveal that the resulting multilayer graphene is of turbostratic stacking. We further propose a growth mechanism of graphene on silver from studies of the X-ray photoelectron spectroscopy (XPS) and cross-sectional annular dark-field scanning TEM (ADF-STEM) and demonstrate that XPS data may be used as a nondestructive means to infer the average graphene thickness in agreement with the ADF-STEM results. Confirmed through XPS, the surface of silver fully covered by the directly grown multilayer graphene exhibit no traces of oxidation after 5 months of ambient air exposure, which is in stark contrast to the XPS data of a controlled silver surface without graphene coverage, implying perfect passivation of silver by the PECVD-grown multilayer graphene. The excellent protection of silver from oxidation provided by PECVD-grown graphene and the improvement of the underlying silver crystallinity after the PECVD process suggests that our approach paves ways toward realistic technological applications of graphene-protected silver electrodes and surfaces as well as hybrid graphene-silver plasmonics.

## 2 Materials and Methods

### 2.1 Substrate Preparation

The Ag substrate in this work included a Si substrate covered with a 100 nm-thick Ti adhesion layer and a 500 nm-thick Ag thin film on top, both deposited via an electron-beam (e-beam) evaporator.

### 2.2 PECVD Graphene Growth

**Figure 1:**
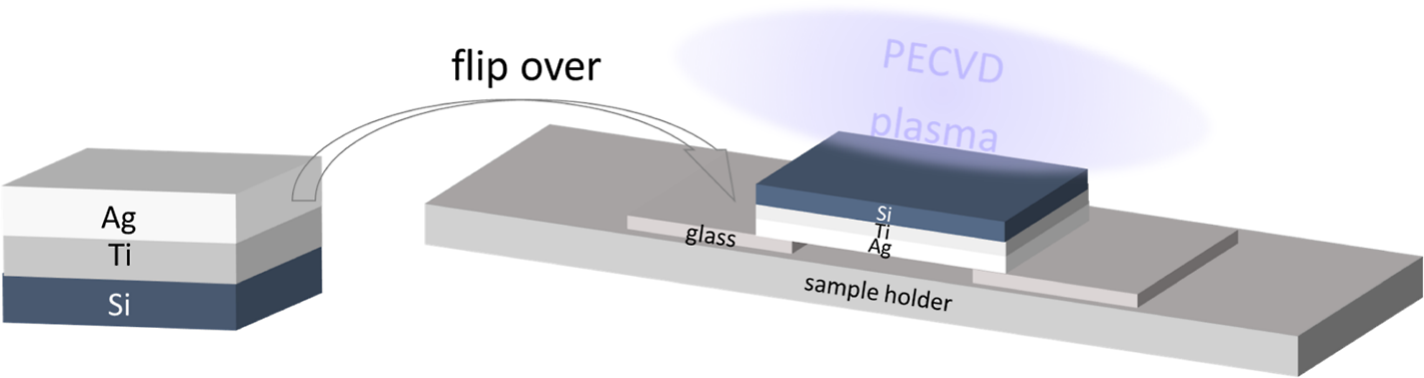
Schematic of flipping over the Ag/Ti/Si substrate and placing it onto the sample holder for direct PECVD-graphene growth on Ag.

Before the PECVD process, the interior of the quartz tube was rinsed with nitric acid to remove potential silver residue, while sample holders were cleaned with piranha solution (H_2_SO_4_:H_2_O_2_ = 3:1 in volume) at room temperature. Afterward, O_2_ and H_2_ plasma were separately used to clean both the quartz tube and the sample holders. The plasma system involved a microwave power source (Sairem) and an Evenson cavity. Prior to plasma ignition, CH_4_ of 1 sccm and H_2_ of 4 sccm were added into the quartz tube with a total pressure of 100 mtorr. The Ag side of the Ag/Ti/Si substrate was flipped downward and placed on the sample holder before the PECVD process as shown in Figure 1.

10 W of plasma power was used along with various growth times. During the PECVD process, the sample was heated only by direct plasma, and the temperature on the sample was between 232 and 260 °C, as measured by a temperature label (Wahl TEMP-RECORDER 101-4V). After the PECVD process, polymethyl methacrylate (PMMA) was spin-coated on the silver samples covered with PECVD-grown graphene, which was followed by silver etching with a gold etchant (TFA, Transene) in order to transfer the graphene grown on silver to a SiO_2_ substrate to evaluate the graphene coverage. Subsequently, acetone was used to remove the PMMA on the transferred graphene sample. For TEM planar view imaging, the PECVD-grown graphene was transferred onto a Cu grid with a Lacey Formvar film.

### 2.3 Characterization

Raman spectroscopic characterizations for graphene growth and quality confirmation were made by using a Raman spectrometer (InVia, Renishaw) equipped with a 514 nm laser. Atomic force microscopy (AFM, Bruker Dimension Icon) with PeakForce tapping mode was used for surface morphology characterization. XPS (Kratos Axis Ultra) with a monochromated Al K_α_ X-ray source and a hemispherical energy analyzer under a pass energy of 10 eV was used for the high-resolution scan, whereas a pass energy of 80 eV was used for X-ray induced Auger spectroscopy (XAES) of the C KLL Auger region. In addition, the XPS signal was used for estimating the graphene thickness, as elaborated in a later section. The instrument work function was calibrated with respect to the Ag 3d_5/2_ signal. Cross-sectional ADF-STEM and TEM plane view images were acquired by aberration-corrected JEOL ARM-200F operated at 200 kV. Selected-area diffraction was performed using JEOL JEM2100F at 200 kV. XRD (Rigaku Smartlab) was performed using Cu K_α_ radiation and a Ge (220) double-bounce monochromator for K_α2_ elimination.

## 3 Results and Discussion

**Figure 2:**
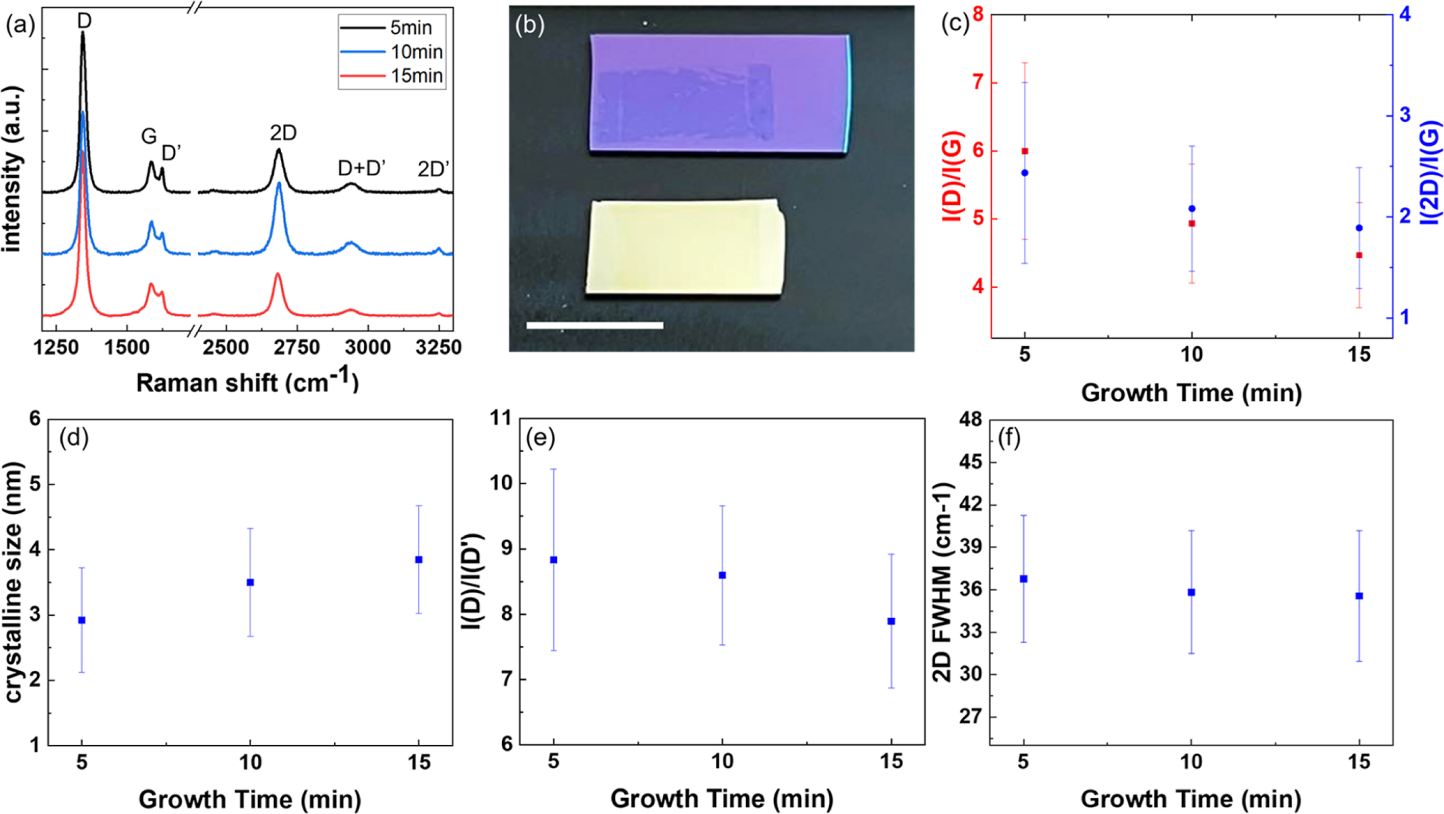
(a) Representative Raman spectrum of the PECVD graphene on silver of different growth times of 5, 10, and 15 min. (b) Graphene transferred onto a SiO_2_ target substrate after its growth. The growth substrate after the removal of graphene was also included side by side with the SiO_2_ target substrate to demonstrate the full coverage of graphene on it before graphene removal. The scale bar is 1 cm. (c) Raman intensity ratios *I*(D)/*I*(G) and *I*(2D)/*I*(G) for samples of different growth times. (d) The graphene grain sizes of different samples extracted from the *I*(D)/*I*(G) ratios and eq 1. (e) *I*(D)/*I*(D′) ratios for samples of different growth times and (f) the full width at half-maxium (fwhm) of the 2D peaks for samples of different growth times.

Figure 2a shows the Raman spectra of PECVD-grown graphene on silver at different growth times. The observed characteristic Raman modes of graphene (i.e., the D, G, D′, 2D, and 2D′ peaks) confirm the successful low-temperature growth of graphene on silver. The optical micrograph image of a SiO_2_ substrate with the transferred graphene as shown in Figure 2b indicates a full coverage of graphene on the silver substrate of (1.0 × 0.7) cm^2^. Interestingly, despite very high *I*(D)/*I*(G) ratios for all samples, the distinct 2D and 2D′ peaks indicated good graphene crystallinity of our PECVD-grown graphene. The 2D and 2D′ peaks of graphene result from intervalley and intravalley phonon scattering, respectively; neither has needs for defect activation.^27,28^ Therefore, one may expect that as the defect concentration increased, 2D and 2D′ peaks would have become worse defined due to imperfect electron dispersion, as demonstrated by Eckmann et al.^29^ In this context, had the high *I*(D)/*I*(G) ratios found in our graphene samples been related to high defect concentrations, distinct 2D and 2D′ peaks would not have existed.

On the other hand, it is known that the grain size of graphene and its defect types may be extracted by analyzing the D-to-G Raman mode intensity ratio [*I*(D)/*I*(G)] and the D-to-D′ Raman mode intensity ratio [*I*(D)/*I*(D′)], respectively.^30,31^ As shown in Figure 2c, the *I*(D)/*I*(G) ratio decreased slightly with increasing growth time, which implied changes in the grain size with growth time. The grain size of graphene *L* can be estimated by the *I*(D)/*I*(G) ratio through eq 1(31)
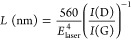
1where *E*_laser_ = 2.41 eV. From eq 1, the graphene grain size was estimated to be ∼3–4 nm, as plotted in Figure 2d. Therefore, the combined observation of clear and distinct 2D and 2D′ peaks and the extracted grain sizes of graphene from the *I*(D)/*I*(G) ratios suggest that the graphene grown on silver thin films via our PECVD method was nanocrystalline. Meanwhile, as shown in Figure 2e, the *I*(D)/*I*(D′) ratio for different growth times was between 8 and 9, suggesting that the defect types were a mixture of sp^3^ bonds for *I*(D)/*I*(D′) = 13 and vacancy defects for *I*(D)/*I*(D′) = 7, although primarily the vacancy type.^29,30^

A common perception in the Raman spectral analysis of graphene is that the 2D to G peak intensity ratio *I*(2D)/*I*(G) may be used for determining the graphene thickness, with *I*(2D)/*I*(G) > 1 implying monolayer graphene. In addition, the full width at half-maximum (fwhm) of the 2D peak may provide further information about the number of graphene layers, with an fwhm of ∼24 cm^–1^ for single-layer graphene^32^ and of ∼50 cm^–1^ for bilayer graphene.^32−34^ However, turbostratic stacking of graphene could also give rise to Raman spectral characteristics similar to those of single-layer graphene. In this context, the *I*(2D)/*I*(G) ratio and the fwhm of the 2D peak for our PECVD-grown graphene on silver shown in Figure 2c,f would imply either single-layer graphene or turbostratic graphene. Therefore, Raman spectral analysis alone appeared insufficient to determine the thickness of graphene conclusively. Additional characterization tools such as TEM and XPS would be necessary to provide a more accurate determination of the graphene thickness, as discussed in the following section.

**Figure 3:**
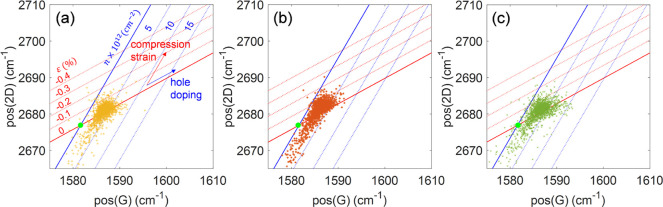
2D peak position “pos(2D)” versus G peak position “pos(G)” of the PECVD graphene on silver samples under growth times of (a) 15, (b) 10, and (c) 5 min, showing slight hole doping and compressional strain in all samples according to the analysis developed by Lee et al.^36^ Here, the light-green solid dot in each panel denotes the strain-free and undoped graphene peak position, and the positive value of the strain corresponds to tensile strain.

Besides the number of graphene layers and the grain size, Raman spectroscopy could also shed light on the doping and strain of graphene. The spectral shift of either the 2D or G peak, along with the corresponding Grüneisen parameter, could be used to extract the strain effect of graphene.^35^ On the other hand, different doping levels of graphene could also lead to a spectral shift of the peak position. As discussed by Lee et al.,^36^ the doping level and strain effect in graphene may be separated by plotting the 2D peak position versus the G peak position. Following a similar analysis, the 2D peak position versus the G peak position of the PECVD-grown graphene on silver showed slightly hole-doped and slightly compressively strained, as demonstrated in Figure 3, where the relevant numbers used for generating the plot coordinates are summarized in Supporting Information Section S1.

**Figure 4:**
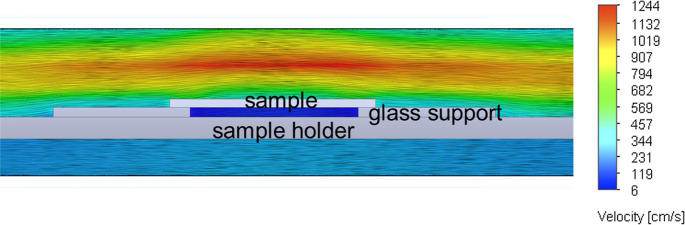
Side view of the gas velocity distribution around the flipped substrate for graphene growth, as obtained by CFD simulations.

To understand the effect of the flipped substrate configuration on the gas flow during the PECVD graphene growth, a computational fluid dynamics (CFD) simulation was carried out. As shown in Figure 4, the gas velocity at the top surface (Si side) of the substrate is significantly higher than that of the bottom side (silver side). Therefore, the benefit of using the flipped substrate configuration is twofold. One is to prevent the energetic plasma from directly damaging the graphene surface. The other is to reduce the gas velocity at the bottom side, which is beneficial to graphene growth by extending the reaction time between the gas species and the substrate.^37^

Figure S2 shows the AFM characterization of the surface morphologies of a sample before and after the PECVD process. After PECVD graphene growth, the AFM image of the surface morphologies was still dominated by the underlying silver layer and revealed a large coalesce of grains and facets, as shown in Figure S2a–c. In contrast, the silver surface before the PECVD process exhibited apparent granular structures, as shown in Figure S2d. The facet formation after the PECVD process may be attributed to the stabilization of graphene on the metal surface.^38^ The increased roughness after PECVD may be attributed to the coalescence of smaller grains, leading to a much larger grain boundary.

Meanwhile, the changes in the crystallinity of silver after the PECVD process, as characterized through XRD measurements, are shown in Figure S3. The increased Ag (111) intensity counts after PECVD for different growth times were all much greater than that before the PECVD graphene growth process. A similar peak intensity for all growth times was observed. Given that no active heating source was involved in our PECVD and that the sample temperature was about 232–260 °C through plasma heating, which was much lower than the melting point of silver (961.8 °C), the improved crystallinity of Ag (111) after the PECVD process when compared to the reference sample may be attributed to the sufficient thermal energy^12^ provided by plasma activation. The improved Ag crystallinity could provide added benefits for silver plasmonic applications.^39,40^

**Figure 5:**
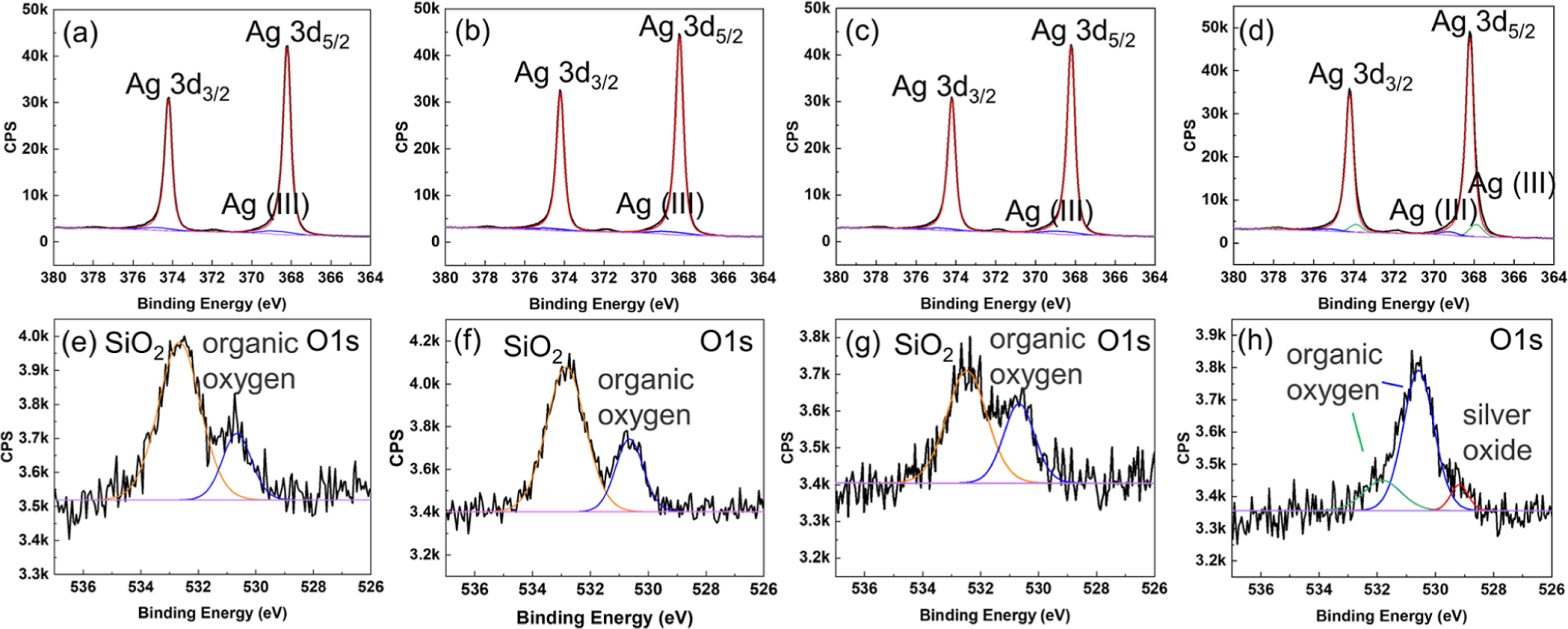
XPS Ag 3d and O 1s spectra taken quickly after the PECVD process for growth times of (a,e) 15, (b,f) 10, (c,g) 5 min, and those of the reference sample (d,h), showing a much-reduced silver oxide component for all samples after the PECVD process.

The chemical changes in the silver thin film quickly after the PECVD process were characterized via XPS as shown in Figure 5. Within the Ag-3d spectrum, the silver oxide component was much reduced after the PECVD process (Figure 5a–c) when compared with the reference sample (Figure 5d). The O 1s spectrum (Figure 5e–h) also supported the observation of reduced metal oxide components. Nevertheless, in the O 1s spectrum, peaks associated with SiO_2_ were also present. The Si 2p region scans were carried out as shown in Figure S4, which confirmed the existence of SiO_2_ on the surface. The origin of the SiO_2_ will be discussed in a later paragraph.

The graphene formation on silver after PECVD was further verified through XPS C 1s spectra as shown in Figure S5, which also confirmed the dominant contribution of sp^2^ carbon. Moreover, neither metal carbide formation^41^ nor bonding between the observed Si and graphene could be inferred from the absence of any apparent peak around the binding energy of 282–283 eV. The extent of hybridization of carbon may be revealed by comparing the “D-parameter” in the C KLL Auger region, where the D-parameter is defined by the peak separation in the first derivative of the Auger spectrum;^42^ a larger D-parameter would indicate a higher sp^2^ hybridization percentage. Using the results from the XAES studies (Figure S6), we obtained the first derivative of the spectra for different growth times shown in Figure S6e–g. In addition to the spectra of different samples before differentiation, the XAES of a graphitic reference has been included in Figure S6d,h to validate the data processing. As shown in Figure S6, the graphene sample with 15 min growth time exhibited the largest *D* value, suggesting a higher sp^2^ percentage than those samples with either 10 or 5 min growth time. This finding also corroborated the lower *I*(D)/*I*(D′) ratio shown in Figure 1, which implied smaller concentrations of sp^3^-like defects.

**Figure 6:**
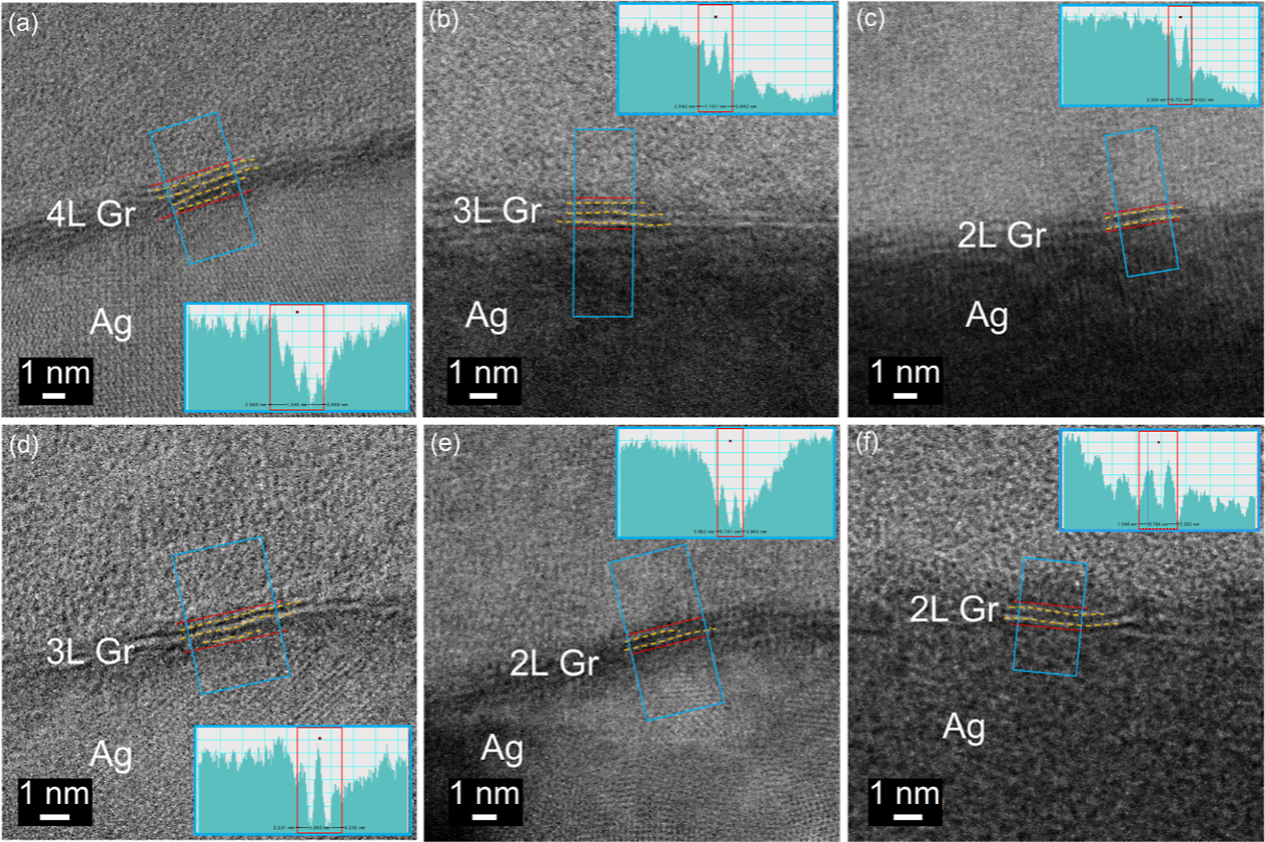
Cross-sectional ADF-STEM images for graphene samples with growth times of (a,d) 15, (b,e) 10, and (c,f) 5 min. The insets showed the averaged intensity profile within the boxed region. The orange dashed lines are for guidance to better reveal the number of graphene layers.

**Figure 7:**
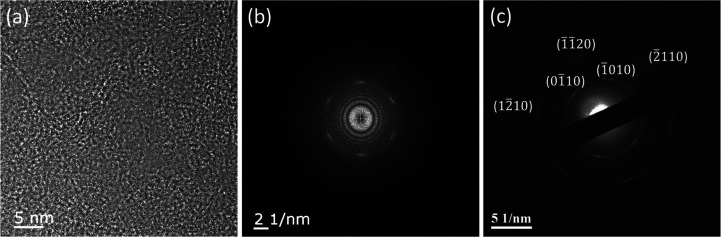
TEM studies of the PECVD-grown multilayer graphene stacking order: (a) TEM image of the planar view of the multilayer graphene sample; (b) FFT of (a); (c) electron diffraction image of the multilayer graphene sample.

ADF-STEM images as shown in Figure 6 provide a direct measure of the number of graphene layers under different PECVD growth times. For the samples with 15 min growth time, we found 3–4 layers of graphene, whereas 2–3 layers for samples with both 10 and 5 min growth times were obtained. To verify the graphene stacking orientation, TEM imaging was performed as shown in Figure 7a. Although the atomic structure was not easily seen, the associated FFT image (Figure 7b) revealed a six-fold symmetry arc pattern instead of discrete spots, suggesting that the angular orientation of graphene layers was random. Using the TEM electron diffraction imaging as shown in Figure 7c, ring patterns and up to second-order diffraction spots were observed. These results further confirmed the turbostratic nature of the multilayer graphene on silver and indicated that our PECVD-grown graphene had good graphene crystallinity despite small grain sizes (or, equivalently, relatively large *I*(D)/*I*(G) ratios in the Raman spectrum).

Comparing the direct imaging of graphene layers by ADF-STEM with XPS characterizations, we investigate whether XPS studies may provide useful information about the average number of graphene layers, similar to previous studies by Hill et al. that proposed to determine the oxide thin film thickness through XPS^43^ and by Cumpson et al. that suggested a “thickogram” graphical approach.^44^ Specifically, the governing equation for the thickogram is given by
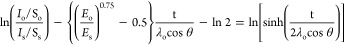
2where *I*_o_ and *I*_s_ are the integrated spectral area under the peaks from the overlayer and the substrate, respectively; S_o_ and S_s_ are the corresponding relative sensitivity factors; *E*_o_ and *E*_s_ are the kinetic energies of the overlayer element (carbon) and substrate element (silver), respectively; λ_o_ is the photoelectron inelastic mean free path, t is the overlayer thickness, and θ is the emission angle. In this work, λ_o_ for graphene was 1.06 nm,^42^ and θ for the XPS system was 0°. Additionally, we note that the XPS spectra used for the thickogram analysis must be recorded with the same number of scans and pass energy.

The advancement of computational power since the graphical approach initially proposed by Cumpson et al. has enabled the numerical computation of sample thicknesses based on the given XPS data. Using the XPS data taken on our PECVD-grown graphene on silver samples with different growth times, we find that the extracted overlayer (i.e., graphene) thicknesses for 15, 10, and 5 min growth times are 1.22, 0.55, and 0.63 nm, respectively, which are in reasonable agreement with the ADF-STEM imaging. Therefore, we have confirmed that XPS data may be used to infer the graphene thickness in addition to Raman spectroscopic analysis and ADF-STEM imaging.

**Figure 8:**
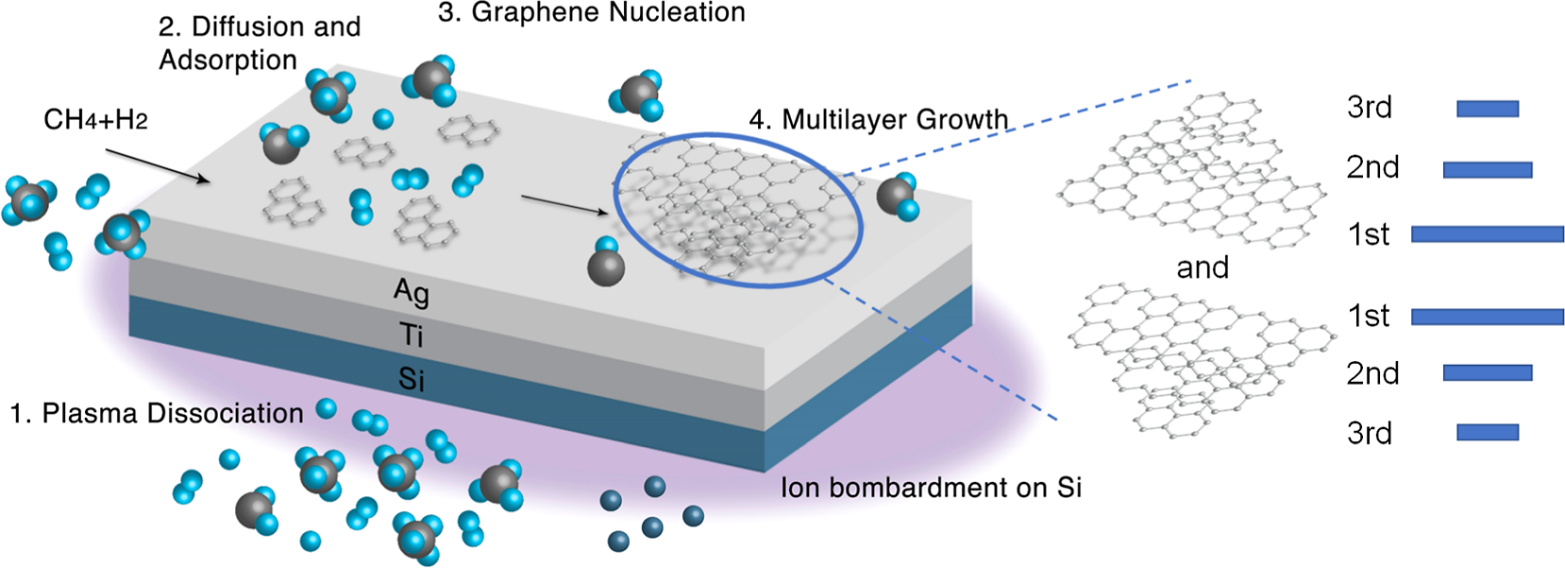
Proposed PECVD graphene growth mechanism on silver.

Based on the XPS and ADF-STEM data, we hypothesize the PECVD graphene growth mechanism on silver, which is schematically shown in Figure 8. Like all other plasma-enhanced deposition processes, our PECVD growth of graphene on silver begins with the creation of energetic radicals and reacting species through the dissociation or excitation of methane and hydrogen by microwave excitation. Additionally, the substrates are heated through direct contact with the plasma. While the plasma is on, some reactive species such as radicals and hydrocarbon species diffuse around the substrate towards the silver side and become adsorbed onto the silver surface and then nucleate into graphene. Meanwhile, the top surface of the substrate that directly faces the plasma (i.e., the Si side) undergoes direct bombardment of energetic ions and radicals in the plasma so that some Si atoms/ions are ejected from the substrate into the plasma, leading to the incorporation of some Si species during the graphene growth. Given the fact that Ag and Au have the same carbon solubility and therefore the same catalytic activity,^46^ the growth mechanism of graphene on silver is expected to be similar to the surface adsorption mechanism on Au. In this context and noting a previous work by Lu et al. on PECVD graphene growth on Au that demonstrated bilayer graphene growth for H_2_/CH_4_ ≥ 1 after 5 min,^20^ our finding of bilayer graphene growth on silver for the same growth time (5 min) with H_2_/CH_4_ = 4 appeared to be consistent with the previous report.

On the other hand, when the growth time was extended to 10 and 15 min, three or four graphene layers were observed. As shown in the ADF-STEM images in Figure 6, additional graphene layers could grow either from the top or beneath the existing graphene layers. This finding suggests that for PECVD graphene growth on silver, multilayer graphene growth could occur not only through the diffusion of the carbon species from graphene edges but also through the adsorption and nucleation of activated carbon and hydrogen species on the existing graphene layer.

It is worth noting that a penetration graphene growth mechanism has been previously proposed by Wu et al.^47^ However, the penetration growth mechanism could not support graphene growth for more than bilayer due to the restricted penetration of carbon atoms,^47^ which contradicts our observation.

Since the graphene growth temperature is dependent on the plasma power, where higher power results in higher temperatures, it is conceivable to apply plasma power greater than 10 W to achieve larger graphene grain sizes with smaller *I*(D)/*I*(G) ratios. However, higher plasma power would lead to more ejected Si species into the plasma and therefore the undesirable result of more Si incorporation into the graphene layers during the growth. This consideration therefore constrains the choice of plasma power during the PECVD growth of graphene on silver.

**Figure 9:**
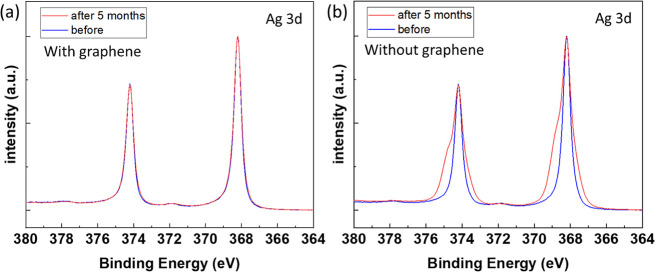
Comparison of the XPS Ag 3d spectra of silver after 5 months of exposure to ambient conditions for (a) a sample fully covered by PECVD-grown graphene and (b) a sample without graphene. Note that the intensity was normalized for better comparison.

An important issue to address for the usefulness of our nanocrystalline multilayer graphene is to evaluate its ability for surface passivation because the small grain sizes are accompanied by many grain boundaries, which may lead to compromised surface passivation because gas molecules could pass through the grain boundaries and react with the underlying silver. Fortunately, we found that the multilayer nature and turbostratic stacking of our PECVD-grown graphene could compensate for the drawback of many grain boundaries. Figure 9 shows the XPS spectra of the Ag 3d region after 5 months of exposure to ambient conditions. There were negligible changes in the peak shape of the silver covered by directly PECVD-grown multilayer graphene. In contrast, the XPS spectra of the silver sample without graphene protection exhibited significant peak broadening and shoulder formation due to oxidation after 5 months. These findings clearly demonstrate that the silver surface was well protected by the multilayer graphene despite of its nanocrystalline size. The excellent passivation may be attributed to the fact that multiple graphene layers with turbostratic stacking could significantly hinder the diffusion pathways of moisture or oxygen molecules from reaching the silver surface.^20,45^

## 4 Conclusions

In conclusion, we report a low-temperature single-step method for direct graphene growth on silver by PECVD for long-term surface passivation. Raman spectroscopic studies of the graphene-on-silver samples suggested that they consisted of nanocrystals with an overall good crystalline quality, underwent a slight compressive strain, and exhibited slight hole doping, with vacancies being the primary defects in the samples. Using CFD simulations, the benefit of using the flipped substrate configuration during the PECVD graphene growth was revealed. From AFM and XRD characterizations, the silver surface morphology and crystallinity after the PECVD process were found to differ from those before the PECVD process, with improved crystallinity after PECVD. The number of graphene layers grown on silver was verified by cross-sectional ADF-STEM images, which varied from 2 to 4 layers depending on the growth time. The stacking order of the multilayer was confirmed through TEM electron diffraction to be turbostratic. We further proposed a mechanism for the PECVD growth of graphene on silver based on the findings from the XPS and ADF-STEM studies and demonstrated that XPS data may be used for the nondestructive thickness determination of graphene. Moreover, the multilayer graphene was found to protect the underlying silver against oxidation for at least 5 months of ambient air exposure. The combined benefits of passivation and improved crystallinity of silver by PECVD-grown graphene imply that our approach paves the way toward scalable technological applications based on graphene-protected silver surfaces and electrodes as well as hybrid graphene-silver plasmonics.

## Supporting Information Available

Relevant parameters used to establish the coordinate in Figure 3; AFM height images of the sample surface after PECVD graphene growth; XRD spectra of the Ag (111) peak taken on samples before the PECVD process and after the PECVD process; Si 2p spectra of PECVD-grown graphene on Ag; XPS C 1s spectra; and XAES C KLL spectra of PECVD-grown graphene on Ag (PDF)
