# Supplementary material for: Low-Temperature Direct Growth of Nanocrystalline Multilayer Graphene on Silver with Long-Term Surface Passivation
Source: ACS Appl Mater Interfaces. 2023 Feb 8;15(7):9883–91. doi: 10.1021/acsami.2c21809 (PMC9951176; doi:10.1021/acsami.2c21809)
Supplement: Supplementary file 1 — am2c21809_si_001.pdf [file am2c21809_si_001.pdf]

Supporting Information

Low-Temperature Direct Growth of Nanocrystalline Multilayer Graphene on Silver with Long-Term Surface Passivation

Chen-Hsuan Lu[a], Kuang-Ming Shang[b], Shi-Ri Lee[c], Chyi-Ming Leu[d], Yu-Chong Tai[b,e] and Nai-Chang Yeh[f,g*]

[a] Department of Applied Physics and Materials Science, California Institute of Technology, Pasadena, California 91125, USA

[b] Department of Medical Engineering, California Institute of Technology, Pasadena, California 91125, USA

[c] Department of Electron Microscopy Development and Application, Division of Platform Technology for Advanced Materials, Material and Chemical Research Laboratories, Industrial Technology Research Institute, Hsinchu 31057, Taiwan

[d] Material and Chemical Research Laboratories, Industrial Technology Research Institute, Hsinchu 31057, Taiwan

[e] Department of Electrical Engineering, California Institute of Technology, Pasadena, California 91125, USA

[f] Department of Physics, California Institute of Technology, Pasadena, California 91125, USA

[g] Department of Physics, National Taiwan Normal University, Taipei, Taiwan

*Email: ncyeh@caltech.edu

Section S1. Relevant parameters used to establish the coordinate of strain and doping plots in Figure 3:

The peak position (G, 2D) for unstrained and undoped graphene is (1581.6, 2676.9) [1]. To generate the strain coordinate, a slope $\left(\frac{\Delta 2D}{\Delta G}\right)_n = 0.7$ was used, whereas a different slope $\left(\frac{\Delta 2D}{\Delta G}\right)_\varepsilon = 2.2$ was used to generate the doping coordinate [1]. Additionally, information about the dependence of either the G or 2D peak on different doping levels was necessary to generate the parallel lines shown in Figure 3 for different strain and doping levels. We used $\frac{\Delta 2D}{\Delta n} = 0.87$ (an average of 0.7 and 1.04 according to Lee, J. E. *et al* [1]) to generate the 2D peak positions for different hole doping levels. The G and 2D peak positions under no strain and with various hole doping levels have been generated using the aforementioned method, as tabulated below in Table S1.

Table S1. G and 2D peak positions with various hole doping levels under no strain

hole doping<br>n x 10^{12} (cm^{-2})	posG (cm^{-1})	pos2D (cm^{-1})
n = 0	1581.600	2676.90
1	1582.843	2677.77
2	1584.086	2678.64
3	1585.329	2679.51
4	1586.571	2680.38
5	1587.814	2681.25
6	1589.057	2682.12
10	1594.029	2685.60
15	1600.243	2689.95

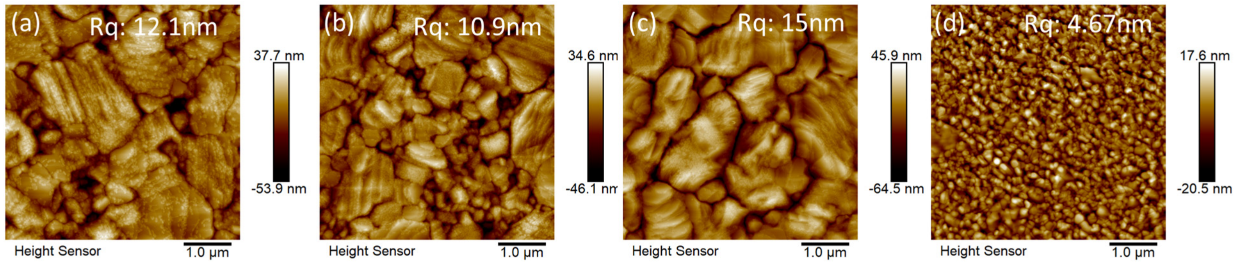

Figure S2. The AFM height images of the sample surface after (a) 15 min, (b) 10 min, and (c) 5 min of PECVD graphene growth, showing significantly increased surface roughness in comparison with (d) for the surface of silver before the PECVD process.

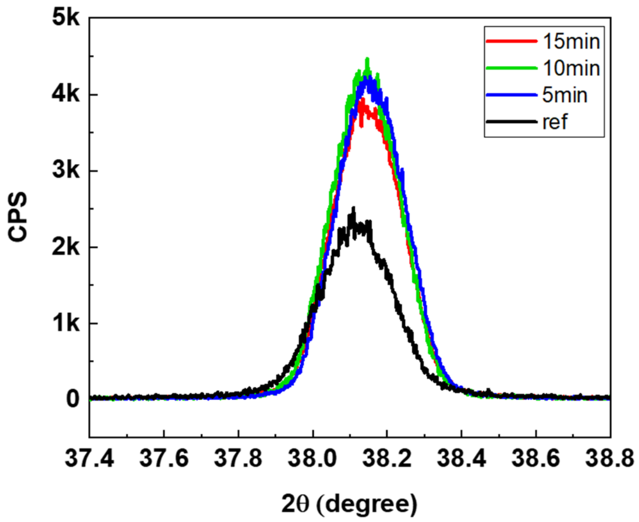

Figure S3. Comparison of the XRD spectra of the Ag (111) peak taken on samples before the PECVD process (denoted as “ref” in the graph) and after the PECVD process for graphene growth over different times of 5 min, 10 min and 15 min.

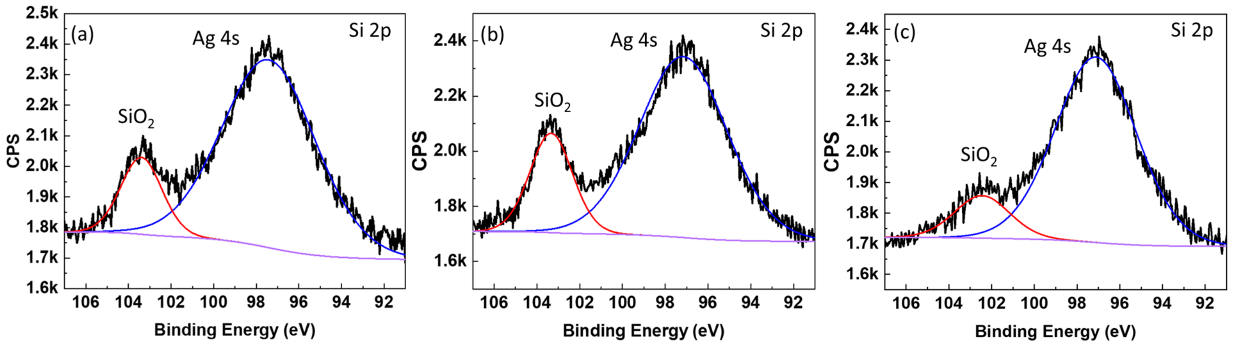

Figure S4. Si-2p spectra of PECVD-grown graphene on Ag after growth times of (a) 15 min, (b) 10 min, and (c) 5 min.

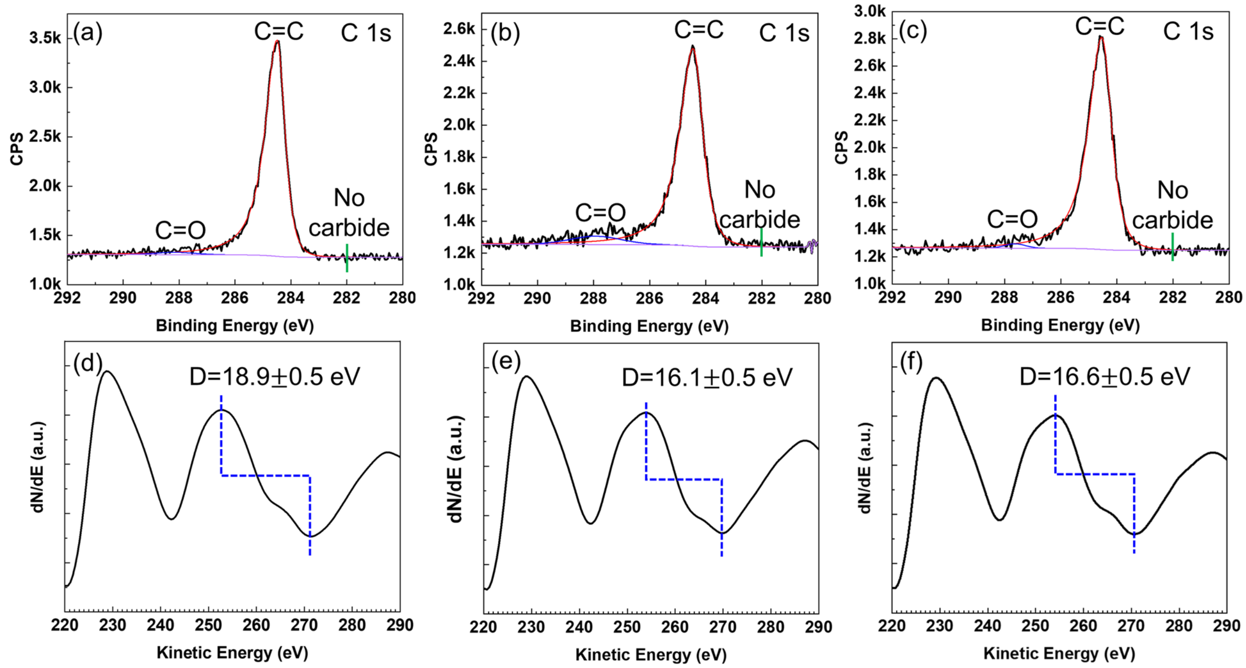

Figure S5. XPS C-1s spectra and the first derivative XAES C KLL spectrum after the PECVD with the growth times of (a,d) 15 min, (b,e) 10 min, and (c,f) 5 min.

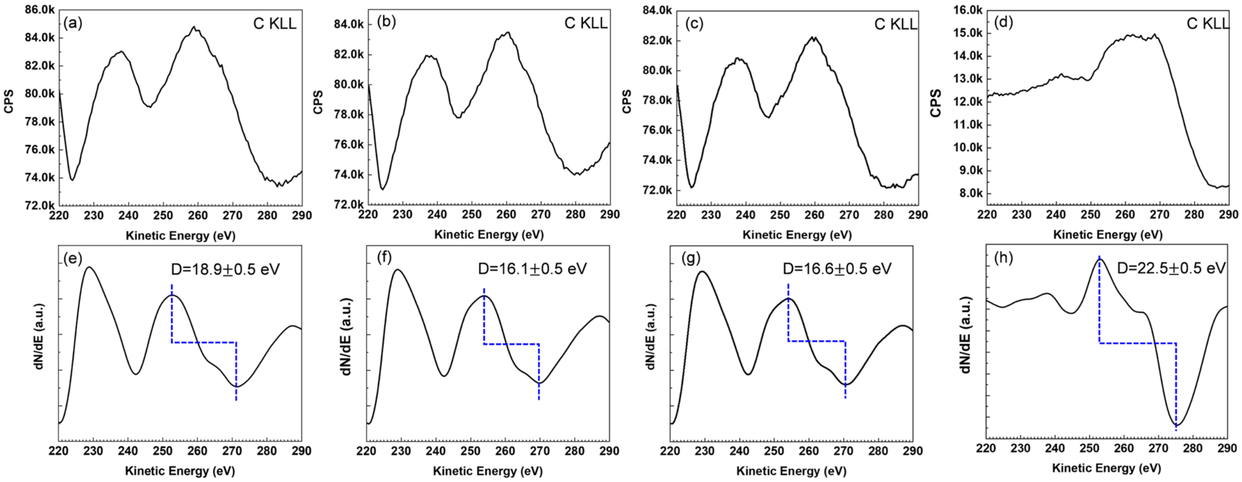

Figure S6. XAES C KLL spectra of PECVD-grown graphene on Ag after growth times of (a) 15 min, (b) 10 min, and (c) 5 min, and (d) the C KLL spectrum of xGnP Graphene Nanoplatelets R10, where R10 was used as the reference graphene material to validate the data processing. (e-h) The first derivative of C KLL spectra of (a-d), respectively. The D value for the graphitic reference sample is in agreement with that in a previous report [2]. Before differentiation, the data were smooth by moving average method with a width of 11 data points. The spectra differentiation was executed with the Savitzky–Golay(SG) quadratic method and a smoothing width of 11 data points using CasaXPS software.

References

(1) Lee, J. E.; Ahn, G.; Shim, J.; Lee, Y. S.; Ryu, S. Optical Separation of Mechanical Strain from Charge Doping in Graphene. *Nat Commun* **2012,** *3*, 1024, DOI: 10.1038/ncomms2022.

(2) Kaciulis, S.; Mezzi, A.; Calvani, P.; Trucchi, D. M. Electron Spectroscopy of the Main Allotropes of Carbon. *Surface and Interface Analysis* **2014,** *46* (10-11), 966-969, DOI: https://doi.org/10.1002/sia.5382.
